# MAIP: a web service for predicting blood‐stage malaria inhibitors

**DOI:** 10.1186/s13321-021-00487-2

**Published:** 2021-02-22

**Authors:** Nicolas Bosc, Eloy Felix, Ricardo Arcila, David Mendez, Martin R. Saunders, Darren V. S. Green, Jason Ochoada, Anang A. Shelat, Eric J. Martin, Preeti Iyer, Ola Engkvist, Andreas Verras, James Duffy, Jeremy Burrows, J. Mark F. Gardner, Andrew R. Leach

**Affiliations:** 1 European Bioinformatics Institute (EMBL-EBI), Wellcome Genome Campus, CB10 1SD Hinxton, Cambridge, United Kingdom; 2 Department of Molecular Design, Data and Computational Sciences, GlaxoSmithKline, Gunnels Wood Road, Hertfordshire SG1 2NY Stevenage, UK; 3 Department of Chemical Biology and Therapeutics, St. Jude Children’s Research Hospital, 262 Danny Thomas Place, Tennessee 38105 Memphis, USA; 4 Novartis Institute for Biomedical Research, 5300 Chiron Way, California 94608- 2916 Emeryville, USA; 5 Hit Discovery, Discovery Sciences, R&D, AstraZeneca, Gothenburg, Sweden; 6 Schrodinger Inc, 120 West 45th Street, 10036-4041 New York, NY USA; 7 Medicines for Malaria Ventures Discovery, 1215 Geneva, Switzerland; 8 AMG Consultants Ltd, Discovery Park House, Discovery Park, Ramsgate Road, CT13 9ND Sandwich, Kent, UK

**Keywords:** Malaria, Antimalarial drug discovery, QSAR, Classification modelling, Open‐source software, Naïve Bayes, Machine learning, Data fusion

## Abstract

Malaria is a disease affecting hundreds of millions of people across the world, mainly in developing countries and especially in sub-Saharan Africa. It is the cause of hundreds of thousands of deaths each year and there is an ever-present need to identify and develop effective new therapies to tackle the disease and overcome increasing drug resistance. Here, we extend a previous study in which a number of partners collaborated to develop a consensus in silico model that can be used to identify novel molecules that may have antimalarial properties. The performance of machine learning methods generally improves with the number of data points available for training. One practical challenge in building large training sets is that the data are often proprietary and cannot be straightforwardly integrated. Here, this was addressed by sharing QSAR models, each built on a private data set. We describe the development of an open-source software platform for creating such models, a comprehensive evaluation of methods to create a single consensus model and a web platform called MAIP available at https://www.ebi.ac.uk/chembl/maip/. MAIP is freely available for the wider community to make large-scale predictions of potential malaria inhibiting compounds. This project also highlights some of the practical challenges in reproducing published computational methods and the opportunities that open-source software can offer to the community.
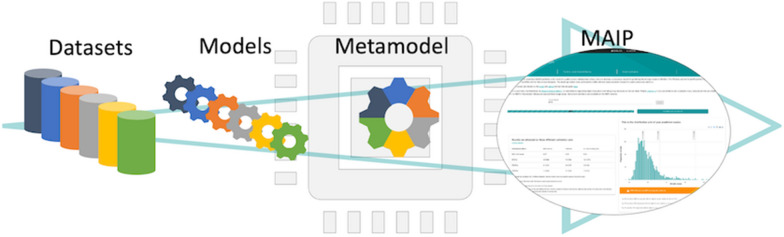

## Introduction

Despite substantial scientific progress, new, affordable and safe malaria medicines are urgently required to overcome increasing resistance against artemisinin-based combination therapies (ACTs), treat vulnerable populations, interrupt the parasite life cycle by blocking transmission to the vectors, prevent infection and target malaria species that transiently remain dormant in the liver. Malaria remains one of the most serious infectious diseases; it threatens nearly half of the world’s population and led to over 400,000 deaths in 2019, predominantly among children in resource-limited areas in Africa, Asia and Central and South America [1].

Clinicians, researchers and, most importantly, patients remain acutely aware of the limitations of the current antimalarial medicines. These include drug resistance, lack of a single dose cure and suboptimal therapies for children and pregnant women. Drug resistance remains a constant threat and patients with resistance to the front-line ACTs are now being routinely identified in Southeast Asia [2]. Secondly, all existing malaria treatments require patient adherence to the course of treatment. A single exposure medicine would allow health workers to directly control and observe drug administration ensuring completion of the treatment course and help to avoid parasite exposure to sub-therapeutic doses [3]. Finally, to protect the entire population, new medicines suitable for use in mothers and babies are urgently needed. Such medicines need age and weight appropriate dosing, child friendly formulation and comprehensive reproductive toxicology evaluation to ensure they are safe to use during pregnancy [4].

The past two decades have seen not only the development of highly efficient high-throughput screening platforms but also significant collaborative efforts between the commercial, not-for-profit and academic communities to join forces in tackling the malaria challenge, often by screening in-house compound libraries and making some of the results available in the literature [5–7]. Whilst precise assay details vary, many of these screens involve cell-based assays that can test for compounds inhibiting the asexual blood cell stage of *Plasmodium spp.* infection, which is the cause of symptomatic malaria. Red blood cells are the site of exponential parasite replication to potentially > 10^12^ parasites per patient, in the case of *Plasmodium falciparum* (PF), and all disease symptoms are caused by the response to the repeated lysis and invasion of erythrocytes by the asexual blood stage parasites. Drug discovery projects starting from the hits arising from these screening experiments have led to the identification of candidates that have been progressed to clinical development. In addition, chemogenomic methods have enabled the discovery of new drug targets and mechanism of action from the same pool of compounds [8, 9].

Machine learning is a computational tool widely-used in drug discovery [10, 11], with its origins in Quantitative Structure-Activity Relationship (QSAR) modelling. Virtual screening is one of the key practical applications of such computational models, enabling large numbers of compounds (including as-yet-unsynthesised molecules) to be assessed for their therapeutic potential prior to experiment [12]. Such machine learning models have the greatest general applicability when trained on large and chemically diverse chemical collections. A practical challenge is that the datasets available for training such models are limited by data availability. Whilst large-scale, open-access databases such as ChEMBL [13] have made a significant impact, nevertheless much bioactivity data is proprietary, making integration a significant challenge due to confidentiality concerns.

Previously, a consortium of Pharma and not-for-profit organisations described a consensus machine learning approach to predict blood stage malaria inhibition [14]. The approach taken involved, first, each partner training a model following the same protocol based on their own in-house data. The models were then shared within the consortium without revealing the confidential information contained in the underlying chemical entities. Proprietary datasets were further obscured by only sharing descriptor weights for the subset of descriptors important in the individual models. A consensus approach, combining several models, showed improved predictivity performance compared to the individual models. The method was subsequently evaluated by selecting and then screening a set of compounds, resulting in a 3-fold enrichment. However, for a number of practical reasons, including the dependence on commercial software, the project was terminated at this stage, and critically, a prediction platform freely available for wider use was not delivered.

Here, we report a successor project, using open-source tools and additional datasets, that has resulted in a freely available and accessible combined model for the community to use in developing new malaria treatments. One of our initial goals was to replicate as closely as possible the original study, whilst using different, open-source software. In addition to the creation of the open-access tool based on a larger dataset, our current study therefore also explores the important, and rarely considered, question of method reimplementation and reproducibility in machine learning for drug discovery [15, 16]. Even with the greater availability of public datasets, there can be dozens if not hundreds of settings that may be required to reimplement the exact same model. Our study may therefore also guide future efforts with regard to what is feasible in this context.

Herein, we report a panel of models trained on blood stage malaria inhibition data from several independent partners using open-source tools. We explore different ways to combine these models in order to achieve enhanced prediction performance. We compare our results with those obtained previously using a variety of measures. We describe our choice of consensus approach that has been implemented in an online prediction platform now freely available for community use.

## Methods

### Training datasets

Eleven datasets from five different partners were used in this study to train models. The Evotec, Johns Hopkins, MRCT, MMV - St. Jude, AZ, GSK, and St. Jude Vendor Library datasets were essentially the same as described previously [14]. The Medicines for Malaria Venture (MMV) partner provided three additional datasets to be used for training models (MMV5, MMV6, MMV7; see Table 1) and the Novartis dataset was significantly different from the original work; these four new or modified datasets are therefore described in detail, as follows:

#### Novartis

The Novartis data set contains 3,355,412 measurements covering 2,726,063 unique canonical smiles assayed in a high-throughput screen using erythrocytes infected with the 3D7 PF strain. The activity cut-off was INH ≥ 50 % at either 1.25 or 12.5 µM compound concentration. Of these, 107,505 always measured as active, 2,593,470 always measured as inactive, and 25,088 were measured ambiguously as both active and inactive. Each measurement was treated as a separate observation in the modelling. The data are proprietary to Novartis and were used to build Novartis models.

#### MMV5

MMV purchased 446,465 compounds that were screened using 3D7 PF strain with SYBR Green dye at 3 µM concentration with an endpoint at 72 h. For the purposes of building a model, compounds were classed as active if they demonstrated an EC50 value < 10 µM or if they showed a mean percentage inhibition at 3 µM > 50 %. On that basis, 4980 compounds were classified as active and 441,485 as inactive. The data are proprietary to MMV and were used to construct MMV5 models.

#### MMV6

MMV purchased 249,444 compounds that were tested using Dd2 PF strain at a concentration of 12.5 µM with a SYBR Green readout. Approximately 8,000 screening actives were selected on the criteria that % inhibition > = 80 %. Then the compounds were clustered and the list reduced to 2,123 compounds that were tested in dose-response on Dd2-SYBR Green with 10 µM top concentration. For the purposes of building a model, compounds tested in the dose-response assay were classed as active if they achieved an EC50 < 10 µM (533 compounds), inactive if not. Of the remainder, compounds were classed as active if their mean percentage inhibition value at 12.5 µM was > = 80 % (inactive if not). This gave a total of 6,328 active and 243,116 inactive compounds for model building. The data are proprietary to MMV and were used to construct MMV6 models.

#### MMV7

MMV purchased 12,732 compounds that were tested at 12.5 µM against Dd2 PF strain using a SYBR Green endpoint at 72 h. 1,073 screening actives were selected based on the criteria that % inhibition > = 70 % in each of 2 replicates. From these, 590 compounds gave a dose-response value < 10 µM. For the purposes of model construction, compounds were considered active if they had an EC50 < 10 µM or if they showed a mean percentage inhibition value of > 80 %. This resulted in 848 active and 11,884 inactive compounds. The data are proprietary to MMV and were used to construct MMV7 models.

### Validation datasets

Three datasets were used for model validation purposes, henceforth referred to as the MMV test set, the PubChem dataset and the St. Jude Screening Set (see Table 1). The latter was already used for model validation in the earlier work but with a single difference due to a compound class change [14].

#### MMV test set

The MMV test set was the outcome of the previous study [14], where the generated consensus model obtained by combining all the partners’ models was used to predict the activity of a collection of 12 million compound extracted from the eMolecules database of commercially available compounds. Compounds with a model consensus score in the − 20 to 0 interval were excluded from further consideration. Substructure search filtering was performed to remove well known antimalarial chemotypes. Only lead-like compounds were selected by using molecular properties, removing potential Pan-assay interference compounds (PAINS) and manual curation. After a clustering step based on the compound molecular fingerprints, 5,869 compounds were tested using PF Dd2 train at 12.5 µM in a blood stage malaria assay by monitoring DNA dye at a MMV Network Test Center. Those with an activity > 50 % were considered as ‘active’ and leading to a total of 1,198 active and 4671 inactive compounds. A resulting threefold hit rate enrichment for the compounds predicted as active relative to the ones predicted as inactive by the model was observed.

#### PubChem

This dataset was obtained combining the results from two assays in PubChem [17].

AID1159554 is a primary screen measuring PF Dd2 strain growth inhibition using SYBR green I in human erythrocytes. 94,441 unique substances were screened at 10 µM and their activity or inactivity status was assigned by PubChem. AID1159566 is a confirmatory screen where 468 substances identified from the primary screen were screened a second time at different concentrations for an EC50 to be determined by regression analysis. Activity and inactivity were also determined directly by PubChem. We combined the results of these assays and considered as active the compounds that were flagged as being ‘active’ in both assays. From this selection, compounds identified as active at a single concentration dose but not validated in the dose-response assay were discarded. Finally, for this validation set 384 compounds were considered as active and the remaining 91,412 were considered inactive.

**Table 1:** Statistics for the datasets used in this study

Dataset origin	Model name	Dataset size	Number of actives	Number of inactives	Ratio of active to inactive compounds
AZ	AZ	11,574	3272	8302	0.3941
GSK	GSK	2,006,390	13,535	1,992,855	0.0068
Evotec	MMV1	229,429	339	229,090	0.0015
Johns Hopkins	MMV2	2,524	247	2,277	0.1085
MRCT	MMV3	40,059	235	39,824	0.0059
MMV - St. Jude	MMV4	305,810	2,507	303,303	0.0083
MMV5	MMV5	446,465	4,980	441,485	0.0113
MMV6	MMV6	249,444	6,328	243,116	0.0260
MMV7	MMV7	12,732	848	11,884	0.0714
Novartis	Novartis	2,700,975	107,505	2,593,470	0.0415
St. Jude Vendor Library	StJudeVendor	541,403	2,026	539,377	0.0038
St. Jude Screening Set	Validation set	220,691	9,082	211,609	0.0429
MMV test set	Validation set	5,869	1198	4671	0.2565
PubChem	Validation set	91,796	384	91,412	0.0042

### Molecule standardisation, descriptor calculation and model-building

The previous study [14] used proprietary methods available via Pipeline Pilot for molecule standardisation, calculation of molecular descriptors and building computational models [18]. For this work we focussed entirely on open-source tools; this would also make it possible for other potential partners to contribute models in the future without limitation.

#### Molecule preparation

We recognise the critical role of data quality in ensuring that the resulting models are as accurate and useful as possible. Due to the particular way that this project was being organised, each partner applied their own in-house data processing, validation and integrity practices. Due to potential differences across the partners in the key question of molecule representation, each molecule was standardised using the same open-source python-rdkit standardiser (available at https://github.com/flatkinson/standardiser) to ensure that the structures were presented in a consistent way to the model-building process. The standardisation involves a number of steps, including identification of the active, “parent” molecule in mixtures and conversion to consistent tautomeric forms. Molecules that failed the standardisation were removed. Descriptors were calculated using RDKit [19] from the standardised structures. Six different combinations of descriptors were explored for each dataset (see Table 2).

**Table 2:** Model descriptor combination for each partner model

Descriptors	ECFP	FCFP	logP	MW	RTB	HBA	HBD	HAC
model 1	X							
model 2		X						
model 3	X		X	X	X	X	X	
model 4	X		X			X	X	X
model 5		X	X	X	X	X	X	
model 6		X	X			X	X	X

*ECFP* unhashed RDKit Morgan fingerprint with radius of 3, useFeatures parameter set to False and useCounts set to True, *FCFP* unhashed RDKit Morgan fingerprint with radius of 3, useFeatures parameter set to True and useCounts set to True, *logP* molecular lipophilicity [20], *MW* molecular weight, *RTB* number of rotatable bounds, *HBA* number of hydrogen bound acceptor, *HBD* number of hydrogen bound donor, *HAC* Heavy atom count

The non-binary descriptors (logP, MW, RTB, HBA, HBD and HAC) were discretised into 10 bins. Unhashed ECFP and FCFP fingerprints were used, so the numbers of bit descriptors varied according to size and chemical diversity of each individual dataset. The resulting data were stored in sparse matrix objects for more efficient processing

#### Model building

A Naïve Bayes approach was used for model building, to ensure consistency with the previous study [14]. This is in general a robust approach for creating models using binary molecular descriptors. We implemented the algorithm described in [21, 22] using the BaseDiscreteNB base class in scikit-learn, to ensure that the model was compatible with all the utilities in the scikit-learn library [23].

In this model, the posterior probability for each feature *Fi* is defined by (1):

1 $$P\left( {active{{|}}Fi} \right) = \frac{{\left( {A_{{Fi}} + 1} \right)}}{{\left[ {T_{{Fi}} \left( {A/T} \right) + 1} \right]}}$$

where $A$ is the number of active molecules in the set, $T$ the total number of molecules in the set, ${T}_{Fi}$ the total number of molecules that contain feature $Fi$ and ${A}_{Fi}$ number of active molecules that contain feature $Fi.$

Variable selection was used to remove uninformative descriptors, defined as those with $abs\left(\text{l}\text{o}\text{g}\right[P\left(active|Fi\right)]$) < 0.05. Finally, the score for a given compound as calculated by the model is defined by (2):

2 $$\begin{array}{l}Pactive = log\left[P\right(active\left|F1\right)] + log[P\left(active\right|F2\left)\right] + ... + log\left[P\right(active\left|Fn\right)]\end{array}$$

### Metrics

#### ROC AUC score

The receiver operator characteristic (ROC) curve is calculated by plotting the fraction of false positives on the x-axis and the fraction of true positives on the y-axis. The area under the curve (AUC) provides a measure of how well a model is able to classify binary data. A value 0.5 corresponds to selecting compounds at random while a perfect model will return a ROC AUC value of 1. This metric is often used to measure the performance of classification models as it is insensitive to class imbalance.

#### Enrichment factor

The enrichment factor (EF) is the hit rate (the proportion of active compounds) within a defined sorted fraction divided by the total hit rate [24]. It is defined by (3):

3 $$\begin{array}{l}EF\left[X\right]=\frac{P\left[X\right]}{N\left[X\right]}.\frac{N}{P}\end{array}$$

where $X$ is the user-defined fraction, $P\left[X\right]$ the number of actives in the fraction, $N\left[X\right]$ the total number of compounds in the fraction, $P$ the total number of actives and $N$ the total number of compounds. The EF is frequently used as a pragmatic measure of performance, reflecting the common use of *in silico* models to identify a subset of compounds for experimental evaluation. In this work, we calculated this metric at 1 % and 10 %, respectively.

#### Fingerprint coverage

The fingerprint coverage compares the set of bit descriptors retained by the Naïve Bayes model with the set of bits derived from the compound fingerprint. Hence, it is calculated as the proportion of bits present in the molecule that are shared with the model [14].

### Software development, model building and sharing

A Dockerfile repository was used to provide a secure and transparent way of distributing the software, containing a json configuration file with the different sets of descriptors for training and the MMV test set for calculating performance metrics. A Python script calculates the molecular descriptors, removes uninformative variables, trains the models and generates performance reports. Two different performance reports are generated; one from a 5-fold cross validation with random splits and the second by training the model on the whole training set and validating it against the MMV test set.

Each partner ran the docker image after having formatted their datasets to fit the platform’s input format and returned to EMBL-EBI all trained models and model performance reports through appropriate secure channels. Models were created in a human readable format, so each partner was also able to verify that no structural or other confidential information was included.

### Data visualisation using t-distributed Stochastic Neighbor Embedding

t-distributed Stochastic Neighbor Embedding (t-SNE) is an algorithm performing a nonlinear dimensionality reduction and designed for data visualisation [25]. The chemical space for the three validation sets was derived from the t-SNE calculation using the same fingerprint descriptors as for model 2 (Table 2). The resulting sparse matrix corresponding to the chemical features present in the validation set compounds was used as input for scikit-learn’s implementation of the t-SNE algorithm using a perplexity value of 500 [23].

## Results

### Validation of model-building protocols

Our first goal was to assess the ability of our new software methods and code to reproduce the previous study [14]. We determined that the major difference would be due to implementations of the descriptor calculations as the distributions of calculated physico-chemical properties are reasonably well (but not perfectly) correlated (Additional file 1: Figure S1). To explore the impact of differences in fingerprint implementations on model-building and performance we used the MMV – St. Jude dataset with only FCFP6 fingerprints and RDKit Morgan fingerprints with radius of 3 and features from Pipeline Pilot and RDKit, respectively. After removing the uninformative bits, we built two Naïve Bayes models and predicted the MMV test set. A pairwise comparison using the Pearson correlation coefficient (R) for the two sets of scores gave a value of 0.88, indicating a good but not perfect correlation (Additional file 1: Figure S2A). In a second comparison, we used ECFP6 fingerprints and RDKit Morgan fingerprints with radius of 3 but without features. This gave an R coefficient of 0.98, indicating almost perfect identity between the two model implementations (Additional file 1: Figure S2B).

These results are not unexpected and are due to differences in the way that which ECFP and FCFP fingerprints are calculated. Both are circular fingerprints, describing the environment in the vicinity of each atom in the molecule. ECFP captures the atom-based substructural information while FCFP represents function-based substructural information [26]. Differences in feature definitions between Pipeline Pilot and RDKit explain the lower correspondence between models built using these two approaches, whilst the ECFP implementations are almost identical.

Nevertheless, the results did confirm to our satisfaction that it would be possible to deliver model performance that was very close to those of the previous study.

### Model comparison

#### Internal validation

**Fig. 1:**
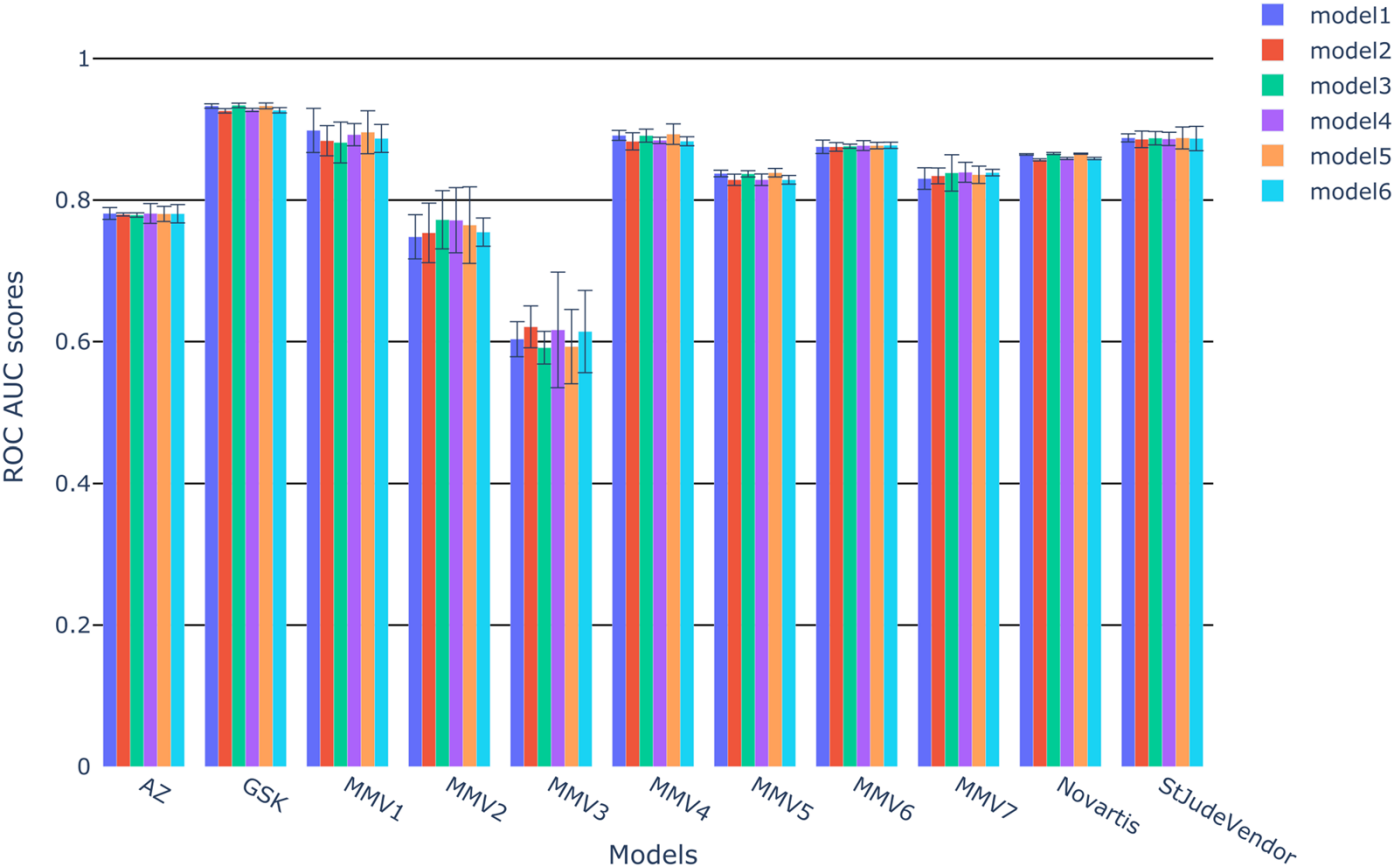
Results of the internal validation for the 6 models trained by each partner dataset. The error bars represent the standard deviation returned by the 5-fold cross validation

The performance for each dataset across the 6 different descriptor combinations is shown in Fig. 1. Somewhat surprisingly, the 5-fold cross validations show that the choice of descriptors appears to have rather limited impact on the performance. Indeed, we observed little variability between the six descriptor sets. Moreover, combining physico-chemical descriptors and molecular fingerprints results in barely any effect (comparing model 1, 3 and 4, and model 2, 5, 6, respectively).

#### External validation

**Fig. 2:**
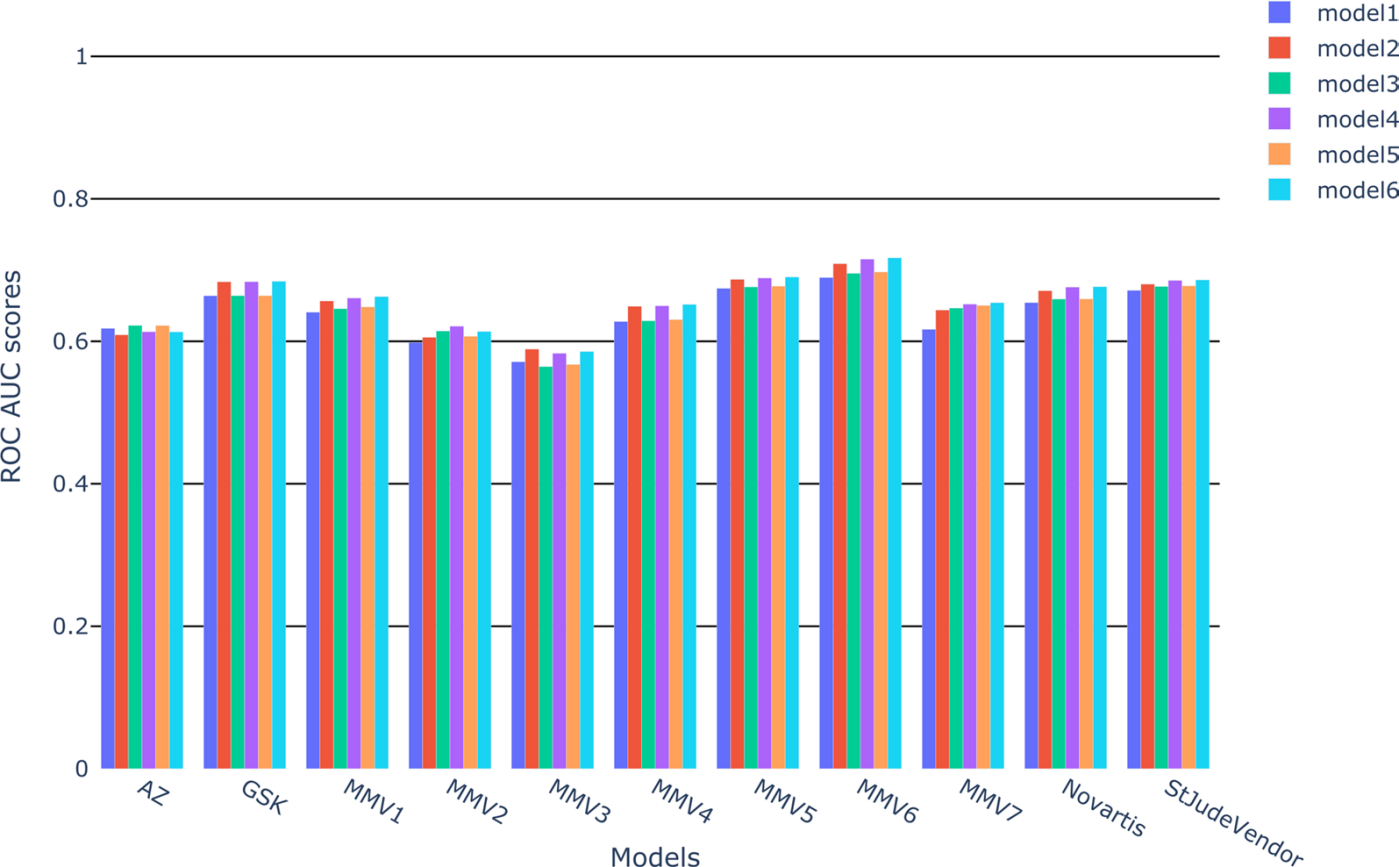
Results of the external validation for the 6 models trained by each partner dataset. This validation was performed predicting the MMV test set

External model performance was evaluated using the MMV test set (Fig. 2). We observed relatively little difference between all the models. When comparing different combinations of descriptors, we observed little variation, though FCFP alone (model 2) is generally better than ECFP alone (model 1).

As the various models were so similar in performance, we pragmatically decided to use the same set of descriptors for each dataset and opted for model 2 (FCFP fingerprints only). Using a single set of descriptors makes the prediction of new compounds more straightforward and faster to compute.

### Consensus approaches

Several consensus options were investigated to identify the optimal approach for inclusion in the public model. We describe here in detail two of the approaches explored, MaxScore and metamodel.

#### MaxScore

**Fig. 3:**
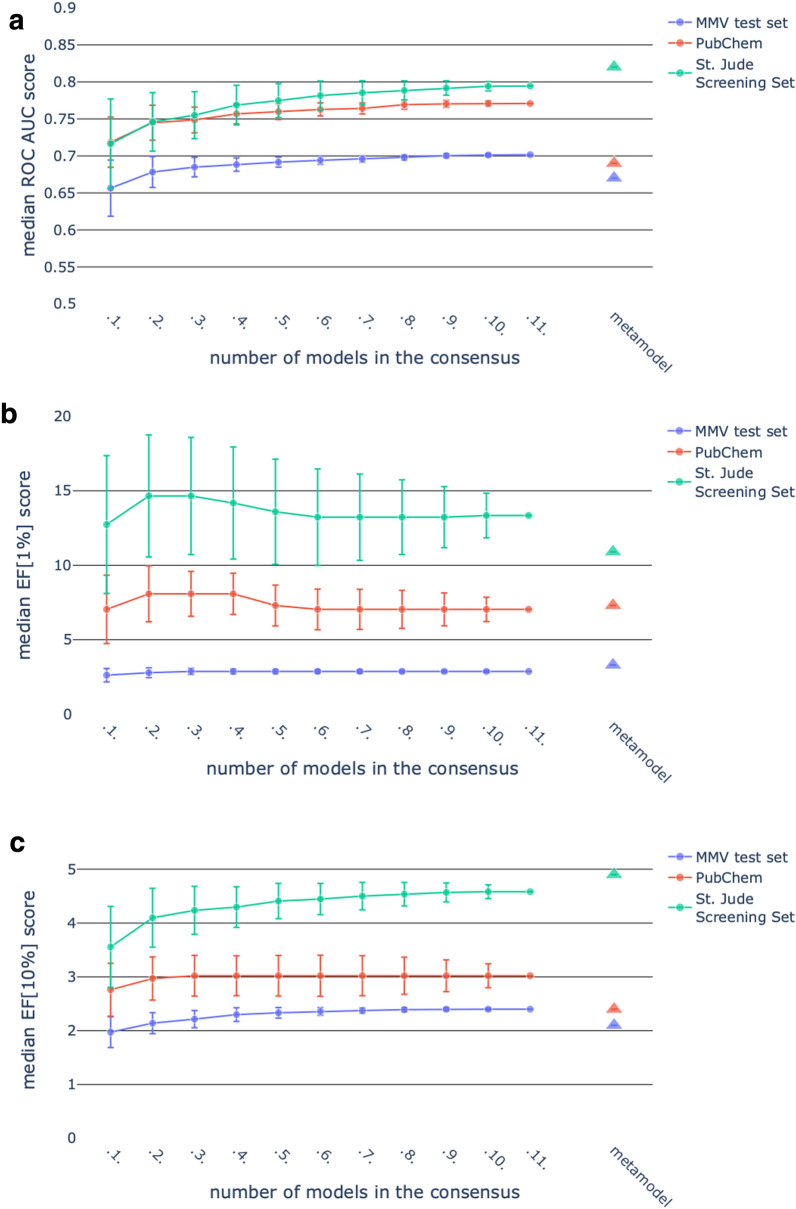
Median performance of the consensus approaches for the MMV (blue), PubChem (red) and St. Jude Screening set (green) validation sets. The error bars represent the standard deviations. The x-axis represents how many models were combined to calculate the MaxScore. For direct comparison, the performance of the metamodel has been added on the right end side of the plots. The performance was assessed by means of **a** the ROC AUC score, **b** EF[1 %] and **c** EF[10 %]

For any given combination of models, the MaxScore prediction for a test compound is the maximum score predicted by any one of the component models. With 11 different models there are a total of 2,047 possible combinations, each containing from 1 to 11 individual models. All 2047 combinations were generated and evaluated using three criteria: ROC AUC score, EF[1 %] and EF[10 %]. EF[1 %] and EF[10 %] represent practical virtual screening scenarios where a small fraction is selected from a pool of compounds. We show in Fig. 3 the impact of increasing the number of models in the MaxScore consensus, using these three metrics for the three validation sets.

Across the three validation sets, we observe significant variance in the performance of individual models; in some cases these outperform the all-model consensus. This is an expected result and it is dependent upon the validation dataset. Also, modest improvements in performance are obtained as more models are included in the consensus for ROC and EF[10 %]. Small consensus models worked best for the EF[1 %] metric for the PubChem and St. Jude Screening sets. The all-model MaxScore consensus delivers a performance at least equivalent to the median performance of other consensus models with fewer contributing models. We also note that enrichment factor performance correlates with the numbers of compounds in the validation sets, with MMV and St. Jude Screening containing the least and most populated ones, respectively. The three validation sets also differ on other aspects. The MMV and St. Jude Screening sets are the results of single-dose assays, while the PubChem active compounds were determined in a dose-response assay. Moreover, the MMV test set is already more enriched with antimalarial compounds than the other validation sets, and was specifically designed in the previous project. This might partly explain its relatively lower enrichment values.

#### Metamodel

In this approach, we merged all the different partner models into a single model based on a combined set of fingerprints bits, where the weight for any one bit is given by summing the weights for that bit across the different individual models. The resulting metamodel is a Naïve Bayes model that generates scores from 893,855 binary variables. It has the key advantage of providing a consensus score from a single model, thus providing significant run-time advantages when compared to the all-model MaxScore consensus which would require each new compound to be first scored by 11 different models. The metamodel approach is compared with the all-model MaxScore consensus in Table 3 and is also annotated on Fig. 3.

In Table 3 we also present summary results for two other consensus approaches that were investigated. The mean score (MeanScore) is calculated as the arithmetic average of the prediction scores returned by the individual models. The MinRank algorithm scores a compound as the minimum rank across all models. Overall, we could not identify a clear winner when comparing the results across these three validation sets.

**Table 3:** Performance comparison between all the consensus approaches implemented, i.e. MaxScore, metamodel, MeanRank and MinRank for the three validation sets

consensus	performance metrics	MMV test set	PubChem	St. Jude Screening Set
MaxScore	ROC AUC score	0.70	0.77	0.79
Metamodel		0.67	0.69	0.82
MeanScore		0.71	0.78	0.80
MinRank		0.64	0.77	0.73
MaxScore	EF[1 %]	2.9	7.0	13.3
Metamodel		3.3	7.3	10.9
MeanScore		2.8	12.8	16.8
MinRank		2.5	7.0	11.0
MaxScore	EF[10 %]	2.4	3.0	4.6
Metamodel		2.1	2.4	4.9
MeanScore		2.3	3.0	4.3
MinRank		1.7	3.0	3.6

### Chemical space analysis

**Fig. 4:**
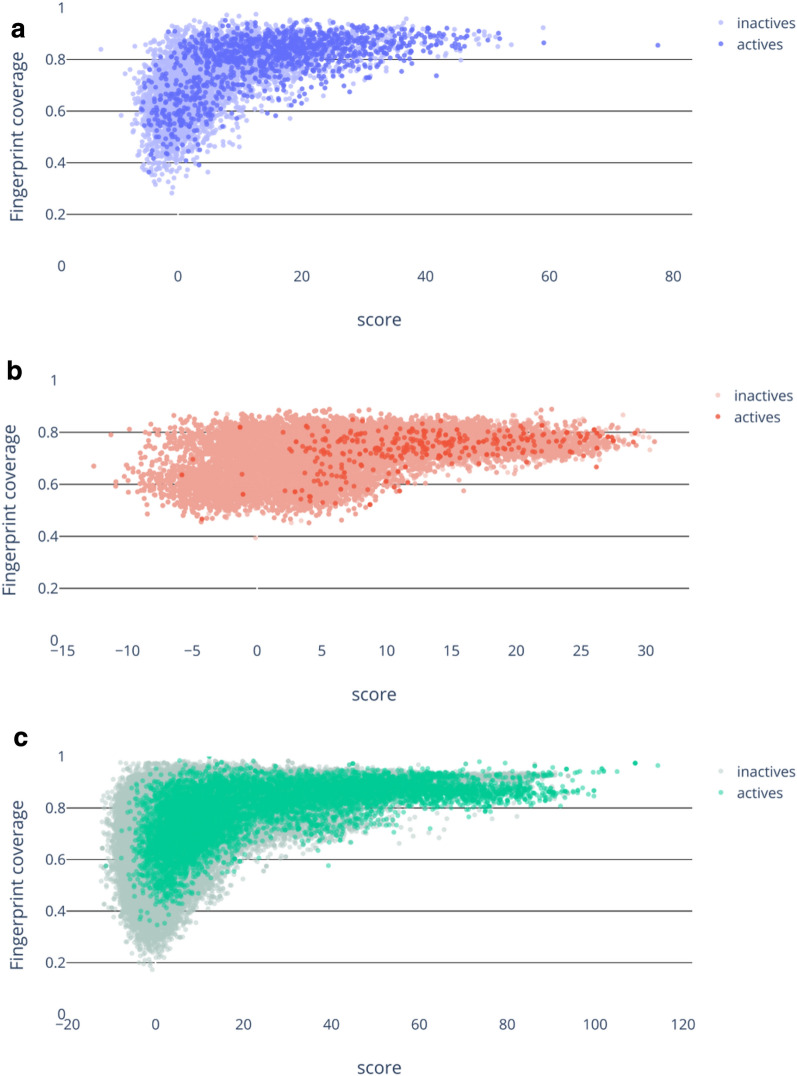
Comparison between fingerprint coverage and prediction score for **a** MMV, **b** PubChem and **c** St. Jude Screening sets, where active compounds are dark and inactive compounds are light

In machine learning a standard way to assess the chemical space over which the model can generate reliable predictions is by defining its domain of applicability [27–29]. It is usually calculated from the variables used to describe the model training set. Methods such as conformal prediction are furthermore able to quantify the prediction certainty [30–32]. Our consensus approaches were developed with the underlying training sets remaining obscured, leaving us with limited options to perform further analysis and in particular it was not possible to directly assess the model’s domain of applicability. Nevertheless, to confirm the predictivity of our methods, we comprehensively assessed their performance using three validation sets as described above. Additionally, we further investigated the variation in performance across these different sets by considering fingerprint coverage. For a given molecule the fingerprint coverage is calculated as the proportion of bits shared with the model and so is a measure of the similarity of the compound with the model. Taking the MaxScore consensus as an example, for each compound in the three validation sets, fingerprint coverage values were calculated as the mean across the 11 models and we compared the resulting metric with the corresponding consensus score, distinguishing active from inactive compounds. See Fig. 4, where active compounds are dark and inactive compounds are light. The MMV and the St. Jude datasets show similar distributions (Fig. 4a and c, respectively), with high compound score for these sets appearing to require high fingerprint coverage. This might be considered expected behaviour since the score is the sum of feature log(probabilities) (see Methods). However, it is not the case that high fingerprint coverage necessarily leads to a high score. Indeed, the metric does not consider the weight of these shared bits, and it is possible that they contribute very little to final score, if not negatively. Having only a few bits shared with the model would be expected to lead to lower scores as there are just too few contributing bits. The results for the PubChem dataset differ from the two others, showing a much weaker relationship between score and the mean fingerprint coverage (Fig. 4b). Interestingly, the lowest fingerprint coverage values in this dataset have higher absolute values than for the other two sets, whilst also having a narrower range of scores.

**Fig. 5:**
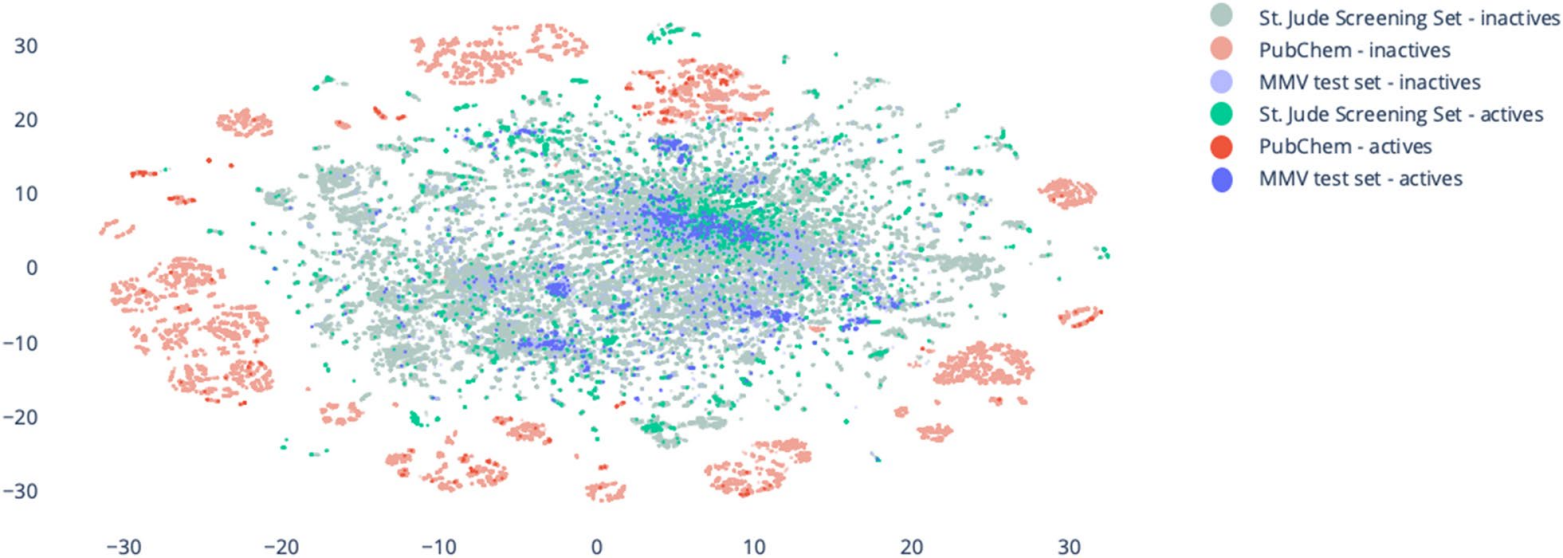
t-SNE visualisation of the three validation set chemical spaces

To further explore this, Fig. 5 shows the results of a t-distributed Stochastic Neighbor Embedding (t-SNE) visualisation of all validation set compounds. Described above, this nonlinear dimensionality reduction technique is particularly useful for preserving local data structures [33, 34]. We observe in Fig. 5 that the MMV test set and St. Jude Screening set compounds are more similar between each other than they are compared to the PubChem dataset compounds, respectively. One possible explanation for these results is that the PubChem set is inherently less diverse, being based around a small number of central scaffolds, than the other two sets. However, such analysis is beyond the scope of this study. We should also be cautious in over-interpreting these results. In particular, the fingerprint coverage metric relies only on the bits shared with the model, ignoring bits in the model but not in the molecule. Hence, while it gives an indication on how much of the compound bits are in the model, it completely eludes the information present in the model but not in the compound. This is analogous to the observations of Martin et al. [35] and Hempel’s “raven paradox”.

### Web application

The above results demonstrate that the metamodel shows comparable results to the other consensus approaches in our validation experiments. Furthermore, there are significant computational advantages in having to deal with just a single model instead of eleven from a system performance perspective. We have therefore implemented the metamodel in a web application called MAIP (MAlaria Inhibitor Prediction), which is available for the community to make antimalarial activity predictions.

**Fig. 6:**
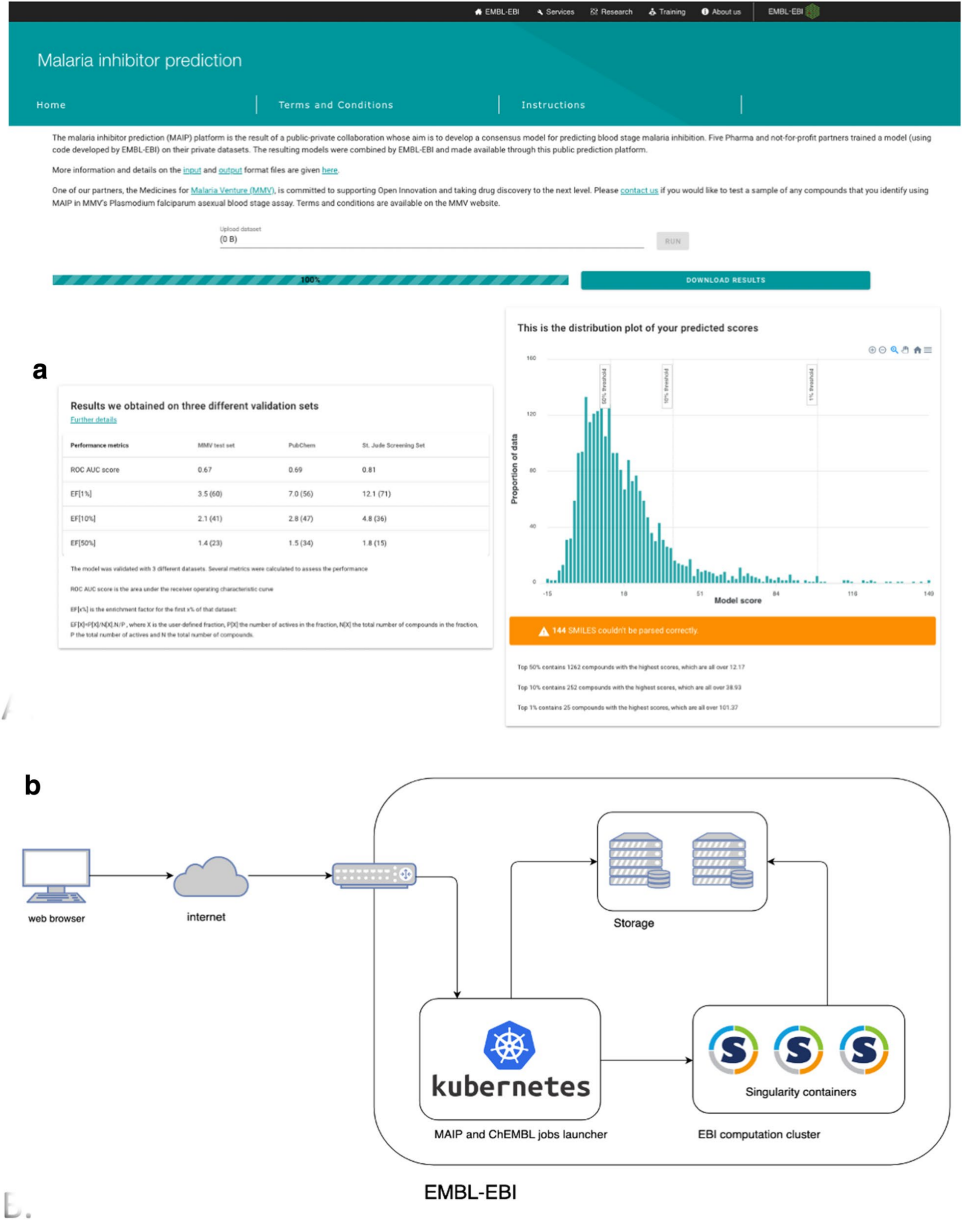
**a** Frontend of the MAIP platform, accessible at https://www.ebi.ac.uk/chembl/maip/; **b** MAIP system architecture

MAIP is accessible through https://www.ebi.ac.uk/chembl/maip/ (Fig. 6a). Users upload a file containing their molecules represented by SMILES strings and associated identifiers. Once submitted, a job is queued for execution on the EMBL-EBI compute infrastructure (Fig. 6b). Each submitted molecule is standardised as described above for consistency with the methodology used for model training, FCFP descriptors are calculated and the molecule is then scored using the metamodel. A file containing individual compound scores and the standardised compound structures can be downloaded when the job finishes. To facilitate analysis of the results, a bar chart giving the score distribution is generated together with summary information on the three validation sets to assist users in any subsequent compound selections. Documentation on the project and MAIP are available at: https://chembl.gitbook.io/malaria-project/.

**Fig. 7:**
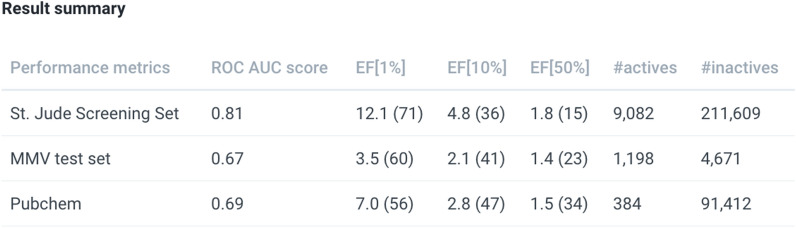
Screenshot from MAIP document page (https://chembl.gitbook.io/malaria-project/using-maip-results#result-summary) showing the model validation results. For each validation set, the numbers of active and inactive compounds are indicated. The scores used to measure the enrichment factors are indicated between parentheses

MAIP is intended to be used as a prioritisation tool, offering users a way to identify compounds of interest for further analysis and selection. MAIP does not directly return a prediction flag, e.g. ‘predicted antimalarial compound’; users should scrutinise their results before making any decisions on next steps. To assist this, documentation is provided with the results together with relevant statistics for our three validation sets (Fig. 7). For each dataset, the higher the model score, the greater the observed enrichment. Further, the thresholds needed to pick 1 %, 10 % and 50 % of the predictions correlate with the dataset size. These data can be used to guide the users in their analysis.

Users are also strongly advised to use additional *in silico* filters to assess the suitability of any virtual hits from MAIP prior to any experimental testing. High scoring compounds may have physicochemical properties and/or substructure features that are unsuitable as starting points for a malaria (or indeed any) drug discovery programme. In addition, some of the training sets used in MAIP contain examples of known antimalarial compounds (e.g. aminoquinolines). Thus, molecules that score highly in MAIP may have already been worked on extensively in antimalarial programmes. Public bioactivity resources such as ChEMBL can be used to determine whether the antimalarial activity is already known for specific structural classes [13]. As part of MMV’s commitment to Open Innovation, screening slots in the MMV *Plasmodium falciparum* asexual blood stage assay are being made available to test 3rd party compounds identified using MAIP. Please contact MMV (maip@mmv.org) for more details.

## Discussion and conclusions

The key output from this work is a practical tool that we hope will be of value in the global search for new malaria treatments. We have built upon and extended a previous study, delivering a computational model derived from > 6.5 million malaria bioactivity values. We believe that this is the largest collection of malaria data assembled to date and the largest public machine learning model for a drug discovery target. We have created a comprehensive open-source software capability for molecule standardisation and model building, based on the widely used RDKit library, that will enable other groups to build their own models and potentially to extend our current model by the inclusion of additional malaria bioassay data. This software was used to deliver 11 separate *in silico* models that individually show good but heterogeneous performance across the three different validation sets employed. We anticipate that this is due to the different chemical space covered by each individual dataset. We cannot exclude the possibility that the validation sets contain compound(s) present in one or more of the 11 training sets as we do not have access to the underlying structures. However, our extensive validation processes demonstrate that the individual models and the consensus model are robust. Furthermore, the consensus models show improved performance and these results also confirm that these approaches are able to deal with data generated with different assay protocols.

Of particular note is that the metamodel approach to combining Naïve Bayes models was shown to deliver good performance across multiple validation sets and also has the key advantage of computational efficiency. This model has been implemented in an easy-to-use web application. Although the compounds used in the training sets remain accessible only to their owners, the wider community can nevertheless take advantage of their malaria bioactivity properties without breaching confidentialities.

MAIP was developed with the objective to be relatively easy to maintain promoting sustainability of the tool. Combined with the open-source software used to develop the model, this will enable both existing and new partners to further contribute to the consensus model. In addition, the modular nature of the MAIP system means that the same approach could be easily applied to other areas, for example with alternative machine learning algorithms and/or to other areas of drug discovery. The scientific community is now invited to use our platform in the hope that this may lead to the initiation of new antimalarial drug discovery projects.

## Supplementary Information

**Additional file 1: Figure S1.** Correlation between physical properties as calculated by Pipeline Pilot and RDKit descriptors on 200k ChEMBL 23 random compounds.** Figure S2.** Correlation analysis between prediction scores returned for the eMolecules validation set by the Pipeline Pilot and the internal models trained with the MMV – St. Jude dataset. (A) Training and validation compounds are described with FCFP6 fingerprints only or RDKit equivalent; (B) Training and validation compounds are described with ECFP6 fingerprints only or RDKit equivalent.

## Data Availability

The code to standardise the compounds and train the models is available on GitHub: standardiser: https://github.com/flatkinson/standardiser. ModifiedNB: https://github.com/chembl/ModifiedNB. mmv_train_image: https://github.com/chembl/mmv_train_image. The ready to use Docker image to generate the models is available at https://hub.docker.com/r/chemblgroup/mmv_train. The MAIP platform is available at https://www.ebi.ac.uk/chembl/maip/.

## Abbreviations

AUC: Area under the curve
ECFP: Extended connectivity fingerprint
EF: Enrichment factor
FCFP: Functional-Class Fingerprints.
HAC: Heavy atom count.
HBA: Number of hydrogen bound acceptor
HBD: Number of hydrogen bound donor
logP: Molecular lipophilicity
MW: Molecular weight
PAINS: Pan-assay interference compounds
PF: *Plasmodium Falciparum*
QSAR: Quantitative structure–activity relationship
RTB: Number of rotatable bounds
t-SNE: t-Distributed Stochastic Neighbor Embedding
