# Supplementary material for: MAIP: a web service for predicting blood‐stage malaria inhibitors
Source: J Cheminform. 2021 Feb 22;13:13. doi: 10.1186/s13321-021-00487-2 (PMC7898753; doi:10.1186/s13321-021-00487-2)
Supplement: Supplementary file 1 — Additional file 1: Figure S1. Correlation between physical properties as calculated by Pipeline Pilot and RDKit descriptors on 200k ChEMBL 23 random compounds. Figure S2. Correlation analysis between prediction scores returned for the eMolecules validation set by the Pipeline Pilot and the internal models trained with the MMV – St. Jude dataset. (A) Training and validation compounds are described with FCFP6 fingerprints only or RDKit equivalent; (B) Training and validation compounds are described with ECFP6 fingerprints only or RDKit equivalent. [file 13321_2021_487_MOESM1_ESM.docx]

|  | **logP** | **MW** | **RTB** | **HBA** | **HBD** |
| --- | --- | --- | --- | --- | --- |
| **R2 score** | 0.7774 | 0.9999 | 0.9713 | 0.9561 | 0.9999 |

Figure S1: Correlation between physical properties as calculated by Pipeline Pilot and RDKit descriptors on 200k ChEMBL 23 random compounds.


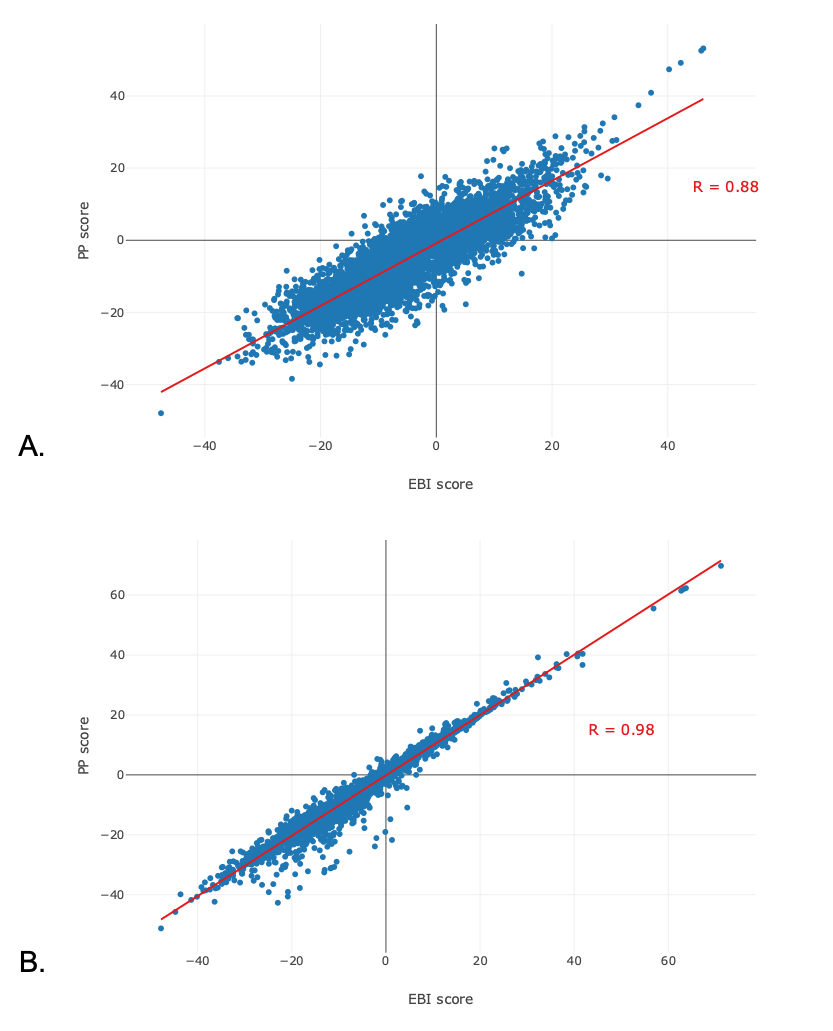


Figure S2: Correlation analysis between prediction scores returned for the eMolecules validation set by the Pipeline Pilot and the internal models trained with the MMV – St. Jude dataset. (A) Training and validation compounds are described with FCFP6 fingerprints only or RDKit equivalent; (B) Training and validation compounds are described with ECFP6 fingerprints only or RDKit equivalent.
